# Effects of Door-to-Door Hang-Up Visits on the Use of Long-Lasting Insecticide-Treated Mosquito Nets in the Democratic Republic of the Congo: A Cluster Randomized Controlled Trial

**DOI:** 10.3390/ijerph18179048

**Published:** 2021-08-27

**Authors:** Paul Mansiangi Mankadi, Yan Jin

**Affiliations:** 1 Environmental Health Department, School of Public Health, University of Kinshasa, BP 127 Kinshasa, Congo; pmansiangi@gmail.com; 2 Department of Microbiology, Dongguk University College of Medicine, Gyeongju 38067, Korea

**Keywords:** long-lasting insecticide-treated mosquito nets, door-to-door hang-up visits, Democratic Republic of the Congo

## Abstract

Malaria accounts for 14% of child deaths in the Democratic Republic of the Congo, and one of the key interventions used to prevent malaria is to distribute insecticide-treated bednets (ITNs), especially long-lasting insecticidal nets (LLINs). The global health community and the Roll Back Malaria initiative have been struggling to achieve universal health coverage using ITNs, and recent studies have reported mixed results about the effects of door-to-door visits and mass distribution campaigns. We aimed to compare LLIN use for those provided by door-to-door hang-up visits and by conventional fixed distribution from distribution centers accompanied by a mass distribution campaign. A cluster randomized control trial was conducted in rural areas of Maniema Province, Democratic Republic of the Congo (DR Congo). Cross-sectional surveys were conducted on 2120 and 2156 households, respectively, with at least one child aged less than five in 76 villages. We assessed the effectiveness of door-to-door hang-up visits on the use of LLINs by exploring the interaction between the “intervention group” and “time” using generalized estimating equation models. Increased LLINs use was observed in all age groups in both arms, but usage differences were not significantly different (relative risk (RR) of LLINs use among children < 5 in the intervention group versus the control group after adjusted for clustering: 1.06, 95% CI: 0.85–1.33). We conclude that the door-to-door hang-up visits are not sufficient to persuade individuals (pregnant woman, children < 5, or all study participants) to use LLINs, although it did appear to be effective for the youngest children in the household.

## 1. Introduction

Malaria remains one of the greatest challenges to the global health community; 409,000 people succumbed to the disease worldwide in 2019 despite substantial efforts made during the past two decades [1]. The Congo is no exception; in 2017, malaria accounted for 14% of child deaths [2]. Apart from its health effects, malaria has enormous economic implications: for example, it reduces labor productivity and increases health expenditures [3].

The distribution of insecticide-treated bednets (ITNs), particularly long-lasting insecticidal nets (LLINs), is one of the key interventions used to prevent malaria [4], and it has been reported that the use of ITNs could reduce under-five child deaths by 14–29% [5,6]. However, high levels of ITN use are critical because of its community-wide herd protective effect [7,8]. To achieve this, the global health community including the Roll Back Malaria initiative has been struggling to achieve universal ITN coverage [9,10,11].

Tremendous efforts have been made to increase the use of LLINs, and these include mass distribution campaigns, which have been shown to be the most effective way of achieving high, equitable coverage and of ensuring the broadest distribution in a short timeframe [12,13,14,15,16,17]. 

The conventional approach to LLIN distribution campaigns is to deliver LLINs to people at a fixed place such as a health center, school, church, mosque, commercial outlet or the like [18]. Therefore, on the basis of recommendations of the World Health Organization (WHO), a new delivery strategy of LLIN distribution emerged, in which community health volunteers (CHVs) hang nets during home visits [18]. 

Recently, the global health community investigated determinants of net use in malaria-endemic areas [13]. Insufficient LLINs in a household to allow coverage of all household members and a lack of willingness to use LLINs have been posited as possible reasons for the difference between LLIN ownership and use [19,20]. Some studies found that social norms and values, power dynamics within families, and physical conditions of nets are key determinants of net use [21,22]. Lack of knowledge and difficulties associated with hanging LLINs have also been suggested as possible barriers to use, whereas some authors concluded difficulties and technical problems associated with net hanging are not significant determinants of non-use [23,24,25,26]. Recent studies have produced mixed results regarding the effectiveness of door-to-door visits on net use [27,28,29]. Thus, there was a need to explore the effects of door-to-door hang-up visits accompanied by a mass distribution campaign using a robust experimental design. 

We hypothesized that the use of LLINs might be increased by applying a door-to-door strategy and LLIN hang-up, that is, installing LLINs on beds, as compared with the usage achieved by fixed distribution campaigns. Before the trial, a pilot study was conducted in 2013 to test this hypothesis in the Musienene and North Kivu Provinces of the DR Congo. This pilot study showed that LLINs were more used when they were distributed using the door-to-door and LLIN hang-up strategy than that achieved by fixed distribution campaigns (unpublished). Based on this result, the National Malaria Control Program, DR Congo suggested that the University of Kinshasa conduct a larger-scale trial to assess the effects of a door-to-door plus hang-up method supported by a mass distribution campaign on the use of LLINs. Accordingly, this study was undertaken in the Congo in order to produce more evidence on the topic. Our objective was to compare LLIN use by those provided with LLINs by the “door-to-door hang-up method” or by conventional “fixed distribution (i.e., delivering LLINs to people at a fixed place such as a health center or the like)” during a mass distribution campaign.

## 2. Materials and Methods

### 2.1. Study Site

The study was carried out in rural areas of the Maniema province, Congo (DR Congo). DR Congo is composed of 26 provinces, and Maniema province has 7 Administrative Territories and an estimated population of 1981,026 distributed over an area of 132,250 km^2^ [30]. Twenty-one ethnic groups including Wazihba (16%), Warega (14%), and Bangubangu (12%) reside in Maniema province [30]. Christianity and Islam are the major religions. The dry season extends from May to August, and the annual average temperature is 25 °C. People largely rely on small-scale agriculture (maize, rice, cassava, banana), livestock, fishing, artisanal mining, or trade. Maniema province is divided into 18 Health Zones. 

### 2.2. Study Design 

#### 2.2.1. Randomization

The study was conducted using a clustered, randomized, controlled design. We used block randomization at the health zone level. 

During the planning stage, we decided that 9 health zones would be targeted using the door-to-door hang-up approach and another 9 would be targeted using conventional methods. This was modified later for a logistical reason, and as a result, the door-to-door hang-up approach was used in 8 health zones and the conventional approach was used in 10. Intervention and control zones were randomly assigned using computer-generated random numbers. 

#### 2.2.2. Random Sampling

For the evaluation, four health zones were randomly selected from 8 intervention and 10 control zones, respectively. There were 57 Health Areas in the four health zones in the intervention and control arms (a total of 114 Health Areas), respectively, and 38 of these Health Areas were randomly selected (19 each in the intervention and control arms). We categorized the villages into two groups (group 1: near, within 5 km from the nearest health center; group 2: far, 5 km or farther from the nearest health center) in each selected Health Area. One village was selected from each category within each selected Health Area; thus, we eventually selected two villages from each Health Area. Villages with <80 households were excluded because we were concerned that we might not be able to survey an adequate number of households having at least one under-five child if the villages size was too small. There was no exclusion criterion for large villages; hence, there was no maximum number of households in this study. Households were selected using the simple random walk method in each village by systematic sampling using a sampling interval. The survey team started from the first household at the main entrance in a village and continued in a clockwise manner to the next household. Households with at least one child aged < 5 years were eligible to participate in the survey. Informed consent was obtained from respondents in written format. In total, 36 villages were selected from each arm. 

### 2.3. Procedure

The mass campaign targeted children under five and pregnant women in all 18 health zones in Maniema province. LLIN distribution was conducted by the Net for Life (NfL) organization (a local NGO) and UNICEF from December 2013 to September 2014. UNICEF conducted an LLIN mass distribution campaign using a conventional strategy in control villages, and NfL implemented a mass distribution campaign in the intervention arm by distributing and fitting LLINs using a door-to-door hang-up visits strategy. Zonal Health Offices facilitated the mass distribution campaign by recruiting community volunteers (called “*recos*” in the province) and supervising nurses in both the intervention and control zones. Health education was conducted in intervention and control communities when distributing LLINs. The *recos* disseminated key messages to household members in the intervention group and at fixed sites (e.g., schools, churches, health centers, and marketplaces) in the control group when distributing LLINs. In the intervention group, the distribution LLINs in each Health Zone took an average of 7 days, which excluded planning, training, and household registration. In the control group, *recos* distributed LLINs for the same period at a fixed place.

In April 2013, a steering committee was established with the remit to assume overall supervision for the project under the direction of the Ministry of Health and the National Malaria Control Program (PNLP). In December 2013, officials from 18 Health Zones and their partners (NFL and UNICEF) developed a detailed plan of action for project implementation, during which the following items were discussed: distribution/storage sites by Health Area; mobilization of personnel for LLIN distribution, household registration and warehouse keeping; transportation; raising awareness of malaria prevention and treatment; and other necessary activities. 

Based on this plan, meetings were held between local authorities (heads Provincial Health Divisions, the provincial manager of the PNLP, team managers of Health Zones, and some representatives of civil society organizations) and community leaders to ensure their support, involvement, and ownership of the project. Overall objectives and key activities of the project were presented, and storage locations and distribution issues concerning the conventional campaign were discussed. Numbers of *recos* required were decided using population sizes, and community population size information was gathered. 

Three types of training were undertaken prior to the distribution of LLINs, that is, at the provincial level, Health Zone, and community volunteer levels. Training was conducted in a cascade-like manner, that is, master trainers of the PNLP trained trainers in the Provincial Health Division and the provincial PNLP. These provincial level trainers trained the 36 officials of the Health Zones and the 146 nurses from both the intervention and health zones. The 36 Health Zone officials included Health Zone medical managers (district chief doctor), administrative managers, and hospital directors. Finally, the 36 officials and 146 nurses trained 292 mobilizers and 3000 *recos*. The *recos* made household visits in the intervention areas. The training for two-thirds of the *recos* (i.e., 2000) were organized by the NfL in the intervention group and the other one-third (i.e., 1000) were organized by UNICEF in the control group; however, the trainers were officials and nurses in both the intervention and control groups. 

*Recos* were trained on how to register households and hang up LLINs and how to provide key messages about malaria prevention and treatment, including the benefits of LLINs. The training content was the same in both intervention and control villages, except for the installation methods of LLINs at the household level. 

*Recos* visited every household in the intervention and control arms and gathered information about each household including the number of household members by gender, pregnant women, children under-five, and beds. While visiting households, they explained the nature of the campaign and their duties. Household data collected by *recos* were compiled at the Health Area level by nurses. After household registration, community volunteers visited households from door-to-door to hang up LLINs directly in sleeping spaces in intervention villages. In control villages, LLINs were distributed under the direction of Health Zone staff, and households were requested to pick up LLINs at specific sites. The campaign was always accompanied by health education regarding the need for behavior changes and the benefits of using LLINs. Education activities were conducted in the intervention and control arms. One LLIN was given to every pregnant woman, one LLIN was allocated to two children per household, and two LLINs were allocated when there were more than two children per household. For other household members, one LLIN was allocated to two persons. The maximum number of LLINs allocated per household was set at five. 

### 2.4. Sample Size

In this study, ownership of LLINs was defined as having at least one LLIN in a household, and use of LLINs was defined as sleeping under LLINs the previous night. Both ownership and use were measured by the respondents’ answers and interviewers’ observations. Accessibility to LLINs in terms of the presence of one LLIN per two people in a household was not considered in the design stage of this trial; this has been described as a limitation of this study in the Discussion.

The primary study outcome was net use among children aged <5 in target areas. We undertook two rounds of cross-sectional surveys using a three-stage cluster sampling method. A baseline survey was conducted before the mass distribution of LLINs, and a second cross-sectional survey was performed 12 months after the campaign started. Seventy-six villages were included in the trial. Households in the 76 villages were re-selected during the second round of the survey. 

The number of villages required for the trial was calculated using an alpha error of 0.05, a study power of 80%, 25 households per village, a non-response rate of 10%, a coefficient of variation of 0.26, and 40% and 50% as proportions of the LLIN use in the absence and presence of the intervention [31]. The total sample size was 2100 in 76 clusters.

### 2.5. Data Collection and Ethical Clearance

**Figure 1:**
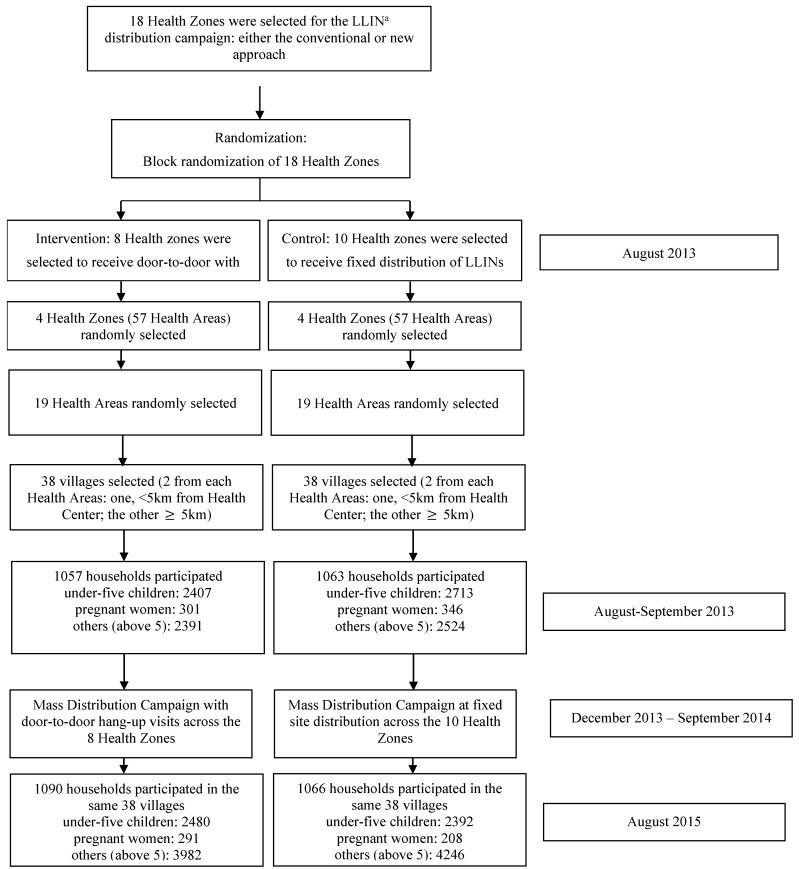
Flow diagram of the trial (^a^ LLIN: long-lasting insecticidal nets).

The flow diagram of this trial is depicted in Figure 1. The baseline survey was done before the mass distribution in 2013. The end-line survey was conducted 12 months after the distribution of mosquito nets in 2015. At baseline and end-line, the questionnaire was completed by 2120 and 2156 households in 76 villages with at least one child aged <5 years.

Forty-eight data collectors were recruited, and all had at least a bachelor’s degree and experience of community health surveys. The data collectors were trained on the survey methodology including questionnaire administration, household selection, and obtaining informed consent over three days. The last day of the training was reserved for a mock-survey exercise. Four supervisors oversaw the survey. Twelve 12 data collectors covered each Health Zone. 

Interviews were conducted with household heads or his/her spouse using structured questionnaires. Data collectors physically observed whether LLINs were hung up as required within households after obtaining written permission from the household head or respondent. Ethical approval for this study was provided by the Institutional Review Board of the University of Kinshasa on September 9, 2013. (ESP/CE/071/13). The study was registered at the International Standard Randomized Control Trial Registry (ISRCTN75108951).

### 2.6. Data Analysis

We assessed effect differences to compare the impacts of the door-to-door hang-up method as compared with conventional fixed-site distribution on the use of LLINs. 

To do so, we explored the interaction between “intervention” and “time” using the generalized estimating equation model. 

The model contained the main effects of intervention and survey time and interactions between these two variables assuming a binomial distribution with an independent correlation structure and log link. Socioeconomic status was also included in the model as a potential confounder, and we adjusted for clustering effect [32]. The baseline values were used to estimate the net effect of the new approach compared with the conventional approach. In addition, because this was an implementation study, we had many stakeholders in this project. Thus, some stakeholders hoped to understand the changes in many specific variables before and after the intervention, rather than looking at the net effect.

## 3. Results

Socioeconomic characteristics at baseline and end-line are shown in Table 1. Briefly, while many characteristics were similar in the intervention and control groups, there were some important differences with respect to respondent sexes both at baseline and end-line and youngest child sexes at baseline. Sociodemographic characteristics showed little change over the study period. The percentages of household heads with completed primary education at baseline and end-line were 48 and 52%, respectively, in the intervention group, and 48 and 44% in the control group. Mean ages of youngest children were similar in both groups at baseline and end-line; mean ages were 18.6 and 18.1 months, respectively, in the intervention group, and 18.6 and 18.3 months in the control group. 

Table 2 shows the effects of household visits with hang-up activities on LLIN coverage for children, pregnant women, and other household members by gender. Before the campaign, the percentage of LLIN use for all household members was 29% in the intervention group and 31% in the control group. The between-cluster coefficient of variation was 0.34 across all 76 villages.

We detected a substantial increase in percentage LLIN use among all ages in both groups over time. For example, LLIN use increased from 48% to 88% during the study period for children aged < 5 years in the intervention group and from 49 to 80% in the control group.

The percentage of LLIN use increased in both groups during the study for all ages, but no significant difference between the arms was observed except for the youngest children. LLINs use by youngest children was higher in the intervention group than in the control group at end-line (Relative risk (RR) adjusted for clustering effect: 1.15, 95% CI 1.03–1.27). Relative risks remained similar after adjusting for clustering, baseline coverage, and confounders. 

Table 3 summarizes knowledge and attitudes to LLINs or malaria and LLIN ownership. A substantial increase in LLIN ownership was evident in both groups. The percentage of households with LLINs increased from 58% at baseline to 94% at end-line in the intervention group and from 60% to 90% in the control group (relative risk (RR) at 12 months 1.09, 95% CI 1.03–1.16). Household members’ knowledge about the cause of malaria, malaria avoidance at home, awareness of LLINs as a means of malaria prevention was already high at baseline. The majority of participants responded that they liked sleeping under LLINs. Children < 5 years old were recognized as the most vulnerable in their households, whereas relatively few people considered pregnant women as most vulnerable. At end-line, people in the intervention group were more likely to perceive that malaria avoidance at home is possible than those in the control group (relative risk (RR) at 12 months 1.17, 95% CI 1.02–1.34). 

## 4. Discussion

We found that LLIN use increased in both the intervention (door-to-door distribution and the hang-up) and control (conventional fixed distribution during a mass distribution campaign) groups. We did not detect a significant difference in percentage LLIN use between the intervention and control groups.

Some previous studies detected no effect of post-campaign home visits on LLIN use. For example, studies in Zambia, Madagascar, and Uganda found that home visits and a distribution campaign had no significant effect on ITN use [27,29,33]. However, a trial in Togo reported a 11.3 to 14.4% increase in LLIN use in intervention communities that were subjected to a distribution campaign and received home visits as compared with control communities [28]. In this previous study, behavior changes were largely attributed to a well-organized media campaign [28]. Our study suggests that intervention had no detectable benefit on LLIN in the intervention group or on children aged < 5 or pregnant women as compared with the control group, although we detected significant increase in LLIN use among youngest children. 

We are reluctant to argue the benefits of home visits versus fixed-point distribution for the following reasons. First, the global strategy for LLINs emphasizes complete coverage of all household members rather than targeting children < 5 and pregnant women [9], and we found no significant benefit of LLIN use among adults, children aged < 5, or pregnant women, which are our primary results. Actually, the significant effect detected for youngest children was a supplementary finding. Second, our study results cast doubt on the advantages of home visits from the perspective of cost-benefit or cost-effectiveness. Although we did not perform benefit/cost analysis, we are pessimistic about the value or benefit conferred of home visits aimed at increasing LLIN use [27]. 

Both agencies used CHVs. The critical difference was that CHVs visited each household to distribute and hang up LLINs in the intervention group, but CHVs distributed LLINs to people at a fixed place in the control group. In both arms, the CHVs delivered the same messages about malaria (prevention and treatment). The only difference was that the CHVs physically visited households to hang up LLINs in the intervention group. 

A previous study suggested that people experiencing a lower incidence of malaria were more likely to perceive malaria avoidance as possible [27,34]. 

In the present study, people in the intervention group were more likely to perceive malaria avoidance at home as possible than those in the control group at end-line. Although we are not sure whether the people in the intervention group would have had a lower incidence of malaria, we infer that this is unlikely to have been the case based on the absence of a significant difference in LLIN use between the two arms. A possible reason for this finding of improved perceptions of malaria avoidance is that the education by CHVs (i.e., delivering messages) at the household level (household by household) might have been much more effective than education provided at a fixed place (delivering messages to a number of people at the same time). If this was the case, repeated hang-up visits by CHVs might increase their LLIN use in the longer term. Another possible reason is courtesy bias, as community residents might have hoped to thank the CHVs for visiting their households by answering this question positively.

The gap between LLIN ownership and use reduced from 9.9% at baseline to 6.2% at end-line in the intervention group and from 11.3% to 9.8%, respectively, in the control group, that is, differences were 3.7% vs. 1.5% in the intervention and control groups, respectively. It was not possible for us to investigate the reason for the increase in net ownership. When designing this study, we assumed that there would be no significant factor affecting net ownership other than the campaign itself in both groups. We also thought the proportion of people purchasing LLINs would not be meaningfully different between before and after the campaign, because encouraging them to purchase LLINs at the market was not highlighted during the campaign. We infer that the increase in net ownership in this study was mainly caused by the LLIN distribution through the campaign (both new and conventional approaches).

In DR Congo, the importance of LLIN use has been emphasized for two decades because malaria is the main killer of children aged under five and pregnant women. The National Malaria Control Program is still struggling to increase LLIN coverages, especially among young children and pregnant women [2]. Somewhat surprisingly, we found many people still do not regard pregnant women as a vulnerable group that requires adequate care. Further study is needed to improve understanding of this misconception and its ramifications. 

This study has several limitations. First, home visits were recorded by *recos* themselves and not verified by respondents. Second, the primary outcome, net LLIN use, was assessed based on respondent recall. Although the likelihood of failing to recall LLIN use during nights before interviews was low, we could not avoid diplomatic or social bias. Although data collectors directly observed LLIN usage in homes to overcome these types of bias, they could not accurately determine whether LLINs were used by the youngest child, a pregnant woman, or any other household member. Third, we should have assessed net possession ratios (i.e., numbers of LLINs per two household members), but we failed to consider this at the design stage. We believe this represents a serious limitation, and one that should be addressed in future studies. An insufficient sample size to detect the effect on the secondary outcome (i.e., the use of LLINs by pregnant women) was another limitation of this study. The heterogeneity in LLIN use and ownership by village based on the actual data was larger than our estimation. The coefficients of variation of LLIN use were 0.41 and 0.35 at baseline and end-line, respectively, and those of ownership were 0.47 and 0.17. Correspondingly, insufficient study power could also be an explanation for the finding of no effect in this study.

A recent study found that the physical quality of LLINs was associated with their use [35,36]. We cannot exclude the possibility that the physical quality of LLINs affected their use differently in the intervention and control group since the campaign period was quite long. We did not survey the physical quality of LLINs, which would be warranted in a future study. The exact question for LLIN use we asked was, “Did you sleep under LLINs last night?” The same questions were asked to mothers (under-5 children), pregnant women, and other household members. Then, if the respondent answered yes, the enumerators made observations inside the sleeping place where the responder slept last night to check that the net was physically hung after obtaining consent for doing so from the respondent. Therefore, we relied on both the results of their responses and enumerators’ observation because we believed that including observational results was very important to reduce bias. However, it was not possible for us to measure use by frequency. More frequent measurements of net use would have been better because longitudinal measurements would have made it possible to investigate trends in net use. 

## 5. Conclusions

In the rural setting of Maniema Province, DR Congo, the door-to-door LLIN hang-up method failed to induce high levels of net use in pregnant woman, children age < 5, or in all study participants, although it seemed to be effective for the youngest children.

We do not argue that it is of no use for CHVs to deliver LLINs through door-to-door visits. Rather, we suggest that caution is needed when selecting a delivery strategy for mass LLIN distribution in terms of cost-effectiveness particularly in resource-limited settings. At the same time, we highlight the finding that the people in the intervention group had more positive perceptions of the preventability of malaria at home after the intervention. This might signal the possibility of improving LLIN use through the door-to-door delivery plus hang-up method, which could be an optimistic aspect of the new delivery strategy that might eventually lead toward universal coverage of LLIN use in the long term. Further research is warranted to obtain more solid evidence of the longer-term effects of the new delivery strategy. 

## Figures and Tables

**Table 1:** Characteristics at baseline and end-line.

Variables	Baseline		End-Line	
	Int ^a^	Con ^b^	Int	Con
	N = 1057	N = 1063	N = 1090	N = 1066
	(Household)	(Household)	(Household)	(Household)
	%/Mean (n/sd)	%/Mean (n/sd)	%/Mean (n/sd)	%/Mean (n/sd)
Child sex of the youngest (male %)	46.6 (493)	53.6 (569)	49.5 (457)	49.6 (487)
Child age of the youngest (months)	18.6 (14.3)	18.6 (13.9)	18.12 (13.43)	18.29 (13.94)
Child sex of all (male, %)	50.0	52.9	50.2	47.7
	(1204/2407)	(1435/2713)	(1246/2480)	(1140/2392)
Child age (months) of all	30.9 (17.3)	31.2 (16.9)	30.1 (16.5)	30.1 (16.8)
Pregnant women age (years)	26.0 (6.6)	25.6 (6.9)	25.2 (8.4)	25.8 (12.7)
Number of pregnant women	301	346	291	208
Respondent sex (male, %)	65.1 (688)	55.0 (585)	57.5 (627)	42.6 (454)
Respondent age (years)	37.0 (12.7)	36.0 (13.3)	37.9 (12.9)	34.0 (11.6)
No of family members	7.9 (4.0)	8.3 (3.5)	8.2 (37.8)	8.1 (3.6)
Household head age	39.7 (13.0)	41.5 (12.8)	42.0 (11.5)	40.9(11.9)
Household head education				
completed primary or uncompleted secondary	47.6	48	51.5	44.1
uncompleted primary	9.7 (35)	12.8 (61)	11.1 (53)	9.2 (62)
completed primary	8.9 (32)	9.6 (46)	8.1 (39)	5.8 (39)
uncompleted secondary	38.7 (139)	38.4 (183)	43.4 (208)	38.3 (258)
completed secondary	24.2 (87)	18.7 (89)	21.9 (105)	24.8 (167)
Respondent education level				
completed primary or uncompleted secondary	45.9	48	48.8	53.2
completed secondary	17.8 (188)	12.1 (129)	14.9 (161)	12.1 (127)
uncompleted secondary	33.6 (355)	37.1 (394)	36.1 (390)	42.4 (446)
completed primary	12.3 (130)	10.9 (116)	12.7 (137)	10.8 (114)
uncompleted primary	21.9 (231)	22.9 (243)	22.0 (238)	22.1 (232)
Link with household head of respondent				
head	66.0 (698)	55.1 (586)	60.6 (659)	42.9 (454)
husband or wife	27.4 (290)	29.1 (309)	29.5 (321)	43.2 (457)
son/daughter	3.9 (41)	10.6 (113)	6.4 (70)	9.4 (99)
Occupation of household head				
Agriculture/Petty traders	87.6 (921)	81.4 (865)	87.2 (951)	81.4 (868)
Civil servant	8.6 (90)	11.5 (122)	8.1 (88)	12.8 (136)

^a^ Intervention, ^b^ Control.

**Table 2:** Primary and secondary outcomes (use of LLINs ^a^ at previous night).

Variables	Baseline		End-Line		Relative Risk	*p*
	Intervention	Control	Intervention	Control		
	% (n/N)	% (n/N)	% (n/N)	% (n/N)		
All children	40.7	38.1	81.0	74.7	1.06 (0.85–1.33)	0.61
	(980/2407)	(1034/2713)	(1983/2449)	(1764/2362)		
Male children	41.5	40.1	81.4	77.5	1.05 (0.82–1.34)	0.72
	(500/1204)	(575/1435)	(995/1223)	(871/1124)		
Female children	39.9	35.9	80.5	72.1	1.05 (0.85–1.31)	0.64
	(480/1203)	(459/1278)	(980/1217)	(892/1237)		
Youngest child	48.1	49.0	87.8	80.2	1.15 (1.03–1.27)	0.01
	(508/1057)	(521/1063)	(957/1090)	(854/1066)		
All household members	28.6	31.2	69.0	63.2	1.24 (0.99–1.55)	0.07
	(667/2330)	(807/2585)	(2846/4125)	(2540/4019)		
Pregnant woman	44.5	39.2	82.5	76.0	1.03 (0.78–1.37)	0.83
	(134/301)	(139/355)	(240/291)	(171/225)		
Female members	32.0	33.1	71.7	66.0	1.30 (0.95–1.77)	0.10
	(376/1176)	(446/1348)	(1527/2131)	(1358/2058)		
Male members	25.2	29.2	66.5	60.4	1.18 (0.98–1.41)	0.07
	(291/1154)	(361/1237)	(1308/1967)	(1177/1948)		

^a^ long-lasting insecticidal nets.

**Table 3:** Intermediate outcomes (ownership, knowledge and attitude to LLINs or malaria).

Variables ^a^	Baseline		End-Line		Relative Risk	*p*-Value
	Intervention	Control	Intervention	Control		
	% (n)	% (n)	% (n)	% (n)		
Having LLIN ^b^	58.0	60.3	94.0	90.0	1.09	0.01
	(645)	(640)	(867)	(881)	(1.03–1.16)	
What causes malaria	88.0	87.6	92.7	85.5	1.12	0.12
	(832)	(930)	(852)	(832)	(0.97–1.31)	
Means of prevention (LLIN) ^c^	92.0	93.1	93.14	90.8	1.06	0.36
	(869)	(989)	(815)	(841)	(0.93–1.21)	
Like sleeping under LLIN	98.6	98.0	99.9	99.6	1.00	0.77
	(931)	(1041)	(908)	(959)	(0.98–1.03)	
The most vulnerable people ^d^: U5C ^e^	43.3	48.6	60.5	58.8	1.12	0.54
	(409)	(514)	(551)	(572)	(0.78–1.61)	
The most vulnerable people ^f^: pregnant women	2.2	1.2	2.2	2.0		
	(21)	(13)	(20)	(19)		
Is it possible to avoid malaria at home?	87.2	93.8	90.1	85.4	1.17	0.03
	(824)	(996)	(815)	(810)	(1.02–1.34)	

^a^ all variables were collected from respondent’s report, ^b^ long-lasting insecticidal nets, ^c^ knowledge of LLINs as a means of preventing malaria, ^d^ recognizing under-five children as the most vulnerable people to malaria, ^e^ under-five children, ^f^ recognizing pregnant woman as the most vulnerable people to malaria.

## Data Availability

Data will be shared upon request (pmansiangi@gmail.com accessed on 11 August 2021).
